# Chromium Biosorption from Cr(VI) Aqueous Solutions by *Cupressus lusitanica* Bark: Kinetics, Equilibrium and Thermodynamic Studies

**DOI:** 10.1371/journal.pone.0137086

**Published:** 2015-09-09

**Authors:** Alma Rosa Netzahuatl-Muñoz, María del Carmen Cristiani-Urbina, Eliseo Cristiani-Urbina

**Affiliations:** 1 Departamento de Ingeniería Bioquímica, Escuela Nacional de Ciencias Biológicas, Instituto Politécnico Nacional, México, D.F., México; 2 Universidad Politécnica de Tlaxcala, San Pedro Xalcaltzinco, Tepeyanco, Tlaxcala, México; 3 Universidad Autónoma de Chiapas, Tuxtla Gutiérrez, Chiapas, México; University of Quebect at Trois-Rivieres, CANADA

## Abstract

The present study investigated the kinetics, equilibrium and thermodynamics of chromium (Cr) ion biosorption from Cr(VI) aqueous solutions by *Cupressus lusitanica* bark (CLB). CLB total Cr biosorption capacity strongly depended on operating variables such as initial Cr(VI) concentration and contact time: as these variables rose, total Cr biosorption capacity increased significantly. Total Cr biosorption rate also increased with rising solution temperature. The pseudo-second-order model described the total Cr biosorption kinetic data best. Langmuir´s model fitted the experimental equilibrium biosorption data of total Cr best and predicted a maximum total Cr biosorption capacity of 305.4 mg g^-1^. Total Cr biosorption by CLB is an endothermic and non-spontaneous process as indicated by the thermodynamic parameters. Results from the present kinetic, equilibrium and thermodynamic studies suggest that CLB biosorbs Cr ions from Cr(VI) aqueous solutions predominantly by a chemical sorption phenomenon. Low cost, availability, renewable nature, and effective total Cr biosorption make CLB a highly attractive and efficient method to remediate Cr(VI)-contaminated water and wastewater.

## Introduction

Environmental chromium (Cr) contamination has become a public health issue because industrial Cr emissions have heavily polluted sites even in close vicinity to residential areas [1]. Among the major Cr pollution sources of aquatic ecosystems are the electroplating and metal finishing industries, iron and steel foundries, the inorganic chemical plants and tanneries [2,3]. Cr contamination has been considered as one of the most serious environmental problems in the last few decades [4]. Furthermore, Cr is considered a priority pollutant in many countries [5].

In aqueous systems, Cr exists primarily in the trivalent [Cr(III)] and hexavalent [Cr(VI)] oxidation states, which have very different physicochemical properties and effects on living organisms [6]. While Cr(III) is relatively immobile due to its low water solubility, Cr(VI) is highly water-soluble and can be transported great distances before being reduced to the trivalent state [7]. In mammals, Cr(III) is an essential trace element required for normal carbohydrate, protein and lipid metabolism [8,9], and is even a popular dietary supplement [10]; however, dosage is important because at high concentrations Cr(III) has adverse effects on cellular structures and functions [11].

Cr(VI), on the other hand, is known to be highly toxic, carcinogenic, mutagenic and teratogenic in mammals, including humans. It can also cause allergic reactions, respiratory disorders, can weaken the immune system, upset the stomach, and bring about kidney, liver and gastric damage [9,12]. Cr(VI) is approximately 100 times more toxic and 1000 times more mutagenic and cytotoxic than Cr(III) [6].

A number of conventional technologies have been used to remove Cr ions from industrial effluents, such as chemical precipitation and filtration, ion exchange, and membrane separation. However, these technologies are either ineffective or expensive when heavy metals are present in low concentrations, or alternatively, when low concentrations of heavy metals in treated waters are required. Besides, they may also generate secondary wastes that are difficult and costly to manage and treat [13].

Among heavy-metal removing technologies, adsorption has gained relevance to treat contaminated aqueous effluents. The most widely used heavy metal-adsorbent is activated carbon; however, its high cost has limited its extensive application in wastewater treatment [14,15]. Furthermore, commercial activated carbon shows low Cr(VI) adsorption capacity [16].

Currently, the trend in Cr(VI) adsorption research is focused on seeking new low-cost adsorbent materials with high adsorption capacity [17–19]. Biological materials have attracted great attention as adsorbents (biosorbents), mainly because they are readily available, and combine excellent adsorption performance and low cost [20,21]. Among those that have been assessed for Cr biosorption from Cr(VI) aqueous solutions are bacteria, yeasts, filamentous fungi and microalgae biomass, by-products and biowastes from the agroindustrial, forestry and fishery industries, substances extracted from biological materials, chemically modified biomaterials, and biological sludge [4,22–24].

However, the removal of Cr(VI) by dead or inactive biological materials is not only challenging because of the biosorption process, but also because, in acidic aqueous solutions, Cr(VI) is reduced to Cr(III) [19,25–30]. At low pH values, part of the Cr(III) generated by Cr(VI) reduction can be found in the aqueous solution; thus, the capacity to remove total Cr of a biomaterial can be lower than its capacity to remove Cr(VI) [27,28,30].

*Cupressus lusitanica* bark (CLB) is abundant, renewable and low-cost, and is capable of removing Cr(VI) from aqueous solutions at different pH levels, both by reducing Cr(VI) to Cr(III) and by Cr biosorption. A kinetic study of total Cr removal by CLB showed that the Cr removal process rate is favored by low solution pH values and that the highest initial biosorption rate is obtained at pH 1.5 [27].

The aim of the present work was to study the effect of relevant environmental parameters, such as initial Cr(VI) concentration, contact time and temperature, on the kinetics of Cr(VI) and total Cr removal by CLB from aqueous solutions. The kinetics of the Cr biosorption process were analyzed using Elovich, fractional power, pseudo-first-order and pseudo-second-order models. The rate constant obtained at different temperatures was used to estimate different thermodynamic activation parameters of the Cr biosorption process. Additionally, total Cr biosorption equilibrium was studied with different initial Cr(VI) concentrations, and the experimental data were modeled with both Langmuir and Freundlich sorption isotherms.

## Material and Methods

### Ethics statement

No specific permits were required for the bark sample collection for this study. We used bark material that came from the private gardens from one of the authors (ARNM). No material was taken from protected land or National Parks. No endangered species were used.

### Biomaterial preparation

*Cupressus lusitanica* bark samples used in this work were collected in the municipality of Panotla, state of Tlaxcala, Mexico. Bark samples were oven-dried at 60°C until they reached constant dry weight. Dried samples were milled using a Glen Creston mill, and the resulting particles were screened using ASTM standard sieves. The fraction with particle size 0.42 to 0.5 mm (meshes 35 and 40) was used in the Cr(VI) and total Cr removal experiments. The sieved biomaterial was stored in an airtight plastic container until used.

### Biosorbent characterization

In order to examine the morphological and surface characteristics of CLB, micrographs of CLB samples were obtained using a scanning electron microscope JEOL, JSM-5800 LV (Japan), at an accelerated voltage of 15 kV after gold coating.

The bulk density, swollen particle density and floatability of CLB were determined following the procedures outlined by Mauguet et al. [31], Volesky [32] and Longhua et al. [33], respectively.

### Kinetic studies of Cr(VI) and total Cr removal

Batch kinetic studies were conducted to evaluate the effect of initial Cr(VI) concentration, contact time and temperature on Cr(VI) and total Cr removal from aqueous solutions by CLB. All experiments were performed in 500 mL Erlenmeyer flasks containing 100 mL K_2_CrO_4_ solution of known concentration and 1 g (dry weight) CLB L^-1^. Throughout the course of the experiments, the pH of each Cr(VI) solution was kept constant at 1.5±0.1 [27] by periodic checking and adjusted with 2 M HCl solution when necessary. Flasks were agitated in an orbital shaker at 100 rpm constant shaking speed.

The effect of initial Cr(VI) concentration on Cr(VI) and total Cr removal by CLB was assessed in Cr(VI) solutions at initial metal concentrations ranging from 10 to 1000 mg L^-1^ (10, 20, 40, 60, 80, 100, 150, 200, 250, 300, 350, 400, 600, 800 and 1000 mg L^-1^), at 28±2°C.

The influence of temperature on the kinetic performance of Cr(VI) and total Cr removal was studied by varying temperatures from 15 to 45±2°C (15, 28, 35, and 45±2°C), and 100 mg L^-1^ initial Cr(VI) concentration.

To check for glass sorption and/or Cr precipitation, CLB-free controls were run simultaneously under exactly the same conditions as the Cr(VI) and total Cr removal experiments. No measurable changes were detected in Cr(VI) and total Cr concentrations, which suggests that the Cr removal observed in the present experiments was only due to the CLB biosorbent.

Samples were collected every 15 min during the first 3 h of contact between CLB and Cr(VI) solution and subsequently at 5, 8, 24, 48 and at least 72 h. Samples were filtered through filter paper (Whatman; grade 42) and the obtained filtrates analyzed for Cr(VI) and total Cr concentrations.

### Equilibrium studies of Cr biosorption

For the equilibrium biosorption experiments, CLB biomass (1 g L^-1^) was mixed with solutions of different initial Cr(VI) concentration (10–1200 mg L^-1^) at pH 1.5 [27], 28±2°C, with constant agitation at 100 rpm for 72 h to ensure biosorption equilibrium was reached. Afterwards, samples were collected, filtered through filter paper (Whatman, grade 42), and then the obtained filtrates were analyzed for total Cr concentration.

### Estimation of Cr biosorption capacity

The amount of total Cr biosorbed at time *t* by the unit mass (dry weight) of CLB or total Cr biosorption capacity (*q*_*t*_, mg g^-1^), was calculated according to the following mass balance equation:

$$q_{t}=\frac{(C_{0}-C_{t})\;V}{W}$$ (1)

where *C*_*0*_ (mg L^-1^) and *C*_*t*_ (mg L^-1^) are the initial and the residual total Cr concentration at time *t*_*0*_ = 0 h and *t* = *t* (h), respectively, *V* is the solution volume (L) and *W* is the dry weight of CLB (g).

### Biosorption kinetics modeling

Information on the kinetics of solute biosorption is crucial to design effective biosorption systems, to select optimum operating conditions for full-scale batch biosorption system and to elucidate the mechanism and potential rate-controlling steps involved in the process of biosorption of metal ions onto the biosorbents [34, 35]. In the present work, the kinetics of the total Cr biosorption data were analyzed using Elovich, fractional power, pseudo-first-order, and pseudo-second-order mathematical models.

#### Elovich model

The Elovich model has been widely applied to chemisorption kinetics data and is often valid for systems in which the adsorbing surface is heterogeneous [36]. This model is expressed as follows [14]:

$$q_{t}=\frac{\text{ln}[\alpha \beta \;t+1]}{\beta }$$ (2)

where *q*_*t*_ is the biosorption capacity (mg g^-1^) at any time *t* (h), *α* is the initial biosorption rate (mg g^-1^ h^-1^), *β* is the desorption constant (g mg^-1^) related to the extent of surface coverage and also to the activation energy involved in chemisorption, and *t* is the contact time (h) between the biosorbent and the metal solution. To simplify the Elovich model, it is assumed that *αβt* >> 1, so the resulting equation is:

$$q_{t}=\frac{\text{ln}[\alpha \beta \;t]}{\beta }$$ (3)

#### Fractional power model

The fractional power model is a modified form of the Freundlich equation and can be expressed as follows [37]:

$$q_{t}=k\;t{\;}^{\nu }$$ (4)

where *v* is the rate constant (h^-1^) and *k* is the constant (mg g^-1^) of the power function model. The product of the power function model constants *kv* can be calculated to obtain the specific biosorption rate at unit time, i.e. when *t* = 1.

#### Pseudo-first-order kinetic model

Lagergren´s pseudo-first-order model is based on the assumption that the rate of biosorption is proportional to the number of free active sites on the biosorbent´s surface. The pseudo-first-order kinetic model is expressed as follows [38]:

$$\frac{dq_{t}}{dt}=k_{1}(q_{e1}-q_{t})$$ (5)

where *q*_*t*_ and *q*_*e1*_ are the biosorption capacities (mg g^-1^) at time *t* (h) and at equilibrium, respectively, and *k*_*1*_ is the rate constant of the pseudo-first-order adsorption (h^-1^).

Integration of Eq (5) with the boundary conditions *t* = 0 to *t* = *t* and *q*_*t*_ = 0 to *q*_*t*_ = *q*_*t*_, gives the following non-linear expression:

$$q_{t}=q_{e1}(1-e^{-k_{1}t})$$ (6)

#### Pseudo-second-order kinetic model

The pseudo-second-order kinetic model assumes that the biosorption rate is controlled by chemical sorption and proportional to the second power of the available fraction of active sites [39]. The pseudo-second-order model is expressed as follows [39]:

$$\frac{dq_{t}}{dt}=k_{2}{\left(q_{e2}-q_{t}\right)}^{2}$$ (7)

where *q*_*e2*_ and *q*_*t*_ are the biosorption capacity (mg g^-1^) at equilibrium and at any time *t* = *t* (h), and *k*_*2*_ is the rate constant of pseudo-second-order biosorption model (g mg^-1^ h^-1^). After integration and applying boundary conditions *t* = 0 to *t* = *t* and *q*_*t*_ = 0 to *q*_*t*_ = *q*_*t*_, the integrated and non-linear form of Eq (7) becomes:

$$q_{t}=\frac{t}{\frac{1}{h}+\frac{t}{q_{e2}}}$$ (8)

where $h=k_{2}q_{e2}^{2}$ is the initial biosorption rate (mg g^-1^ h^-1^).

### Equilibrium modeling

The equilibrium distribution of Cr ions between the aqueous phase and CLB biomass was expressed in terms of a Cr biosorption isotherm. The Langmuir and Freundlich isotherm models, which have been the most widely used models to analyze data for water and wastewater treatment applications, were used in the present work to analyze the experimental equilibrium data of Cr biosorption.

The Langmuir isotherm model is based on the following assumptions: 1) all the adsorption sites are identical, 2) each adsorption site can retain one molecule of adsorbate and consequently the adsorption is limited to monolayer coverage, 3) all sites are energetically and sterically independent of the adsorbed quantity, and 4) the adsorptive forces are similar to the forces in the chemical interaction [38,40]. The Langmuir equation is expressed as follows:

$$q_{e}=\frac{Q_{0}\;b\;C_{e}}{1+b\;C_{e}}$$ (9)

where *q*_*e*_ is the adsorption capacity at equilibrium (mg g^-1^), *Q*_*o*_ is the maximum adsorption capacity, also called the saturated monolayer adsorption capacity (mg g^-1^), *C*_*e*_ is the liquid phase concentration of adsorbate at equilibrium (mg L^-1^), and *b* (L mg^-1^) is the adsorption equilibrium constant (Langmuir constant) which is related to the adsorption energy and quantitatively reflects the affinity between the sorbent and the sorbate [22].

To elucidate whether the biosorption of Cr ions by CLB is favorable or not, the dimensionless separation factor or equilibrium parameter (*R*_*L*_), was determined. The parameter *R*_*L*_ is indicative of the isotherm shape and nature of the sorption process, and is defined as follows [41]:

$$R_{L}=\frac{1}{1+b\;C_{0}}$$ (10)

where *C*_*o*_ is the initial metal concentration (mg L^-1^). The value of this parameter indicates whether the isotherm is unfavorable (*R*_*L*_ > 1), linear (*R*_*L*_ = 1), favorable (0 < *R*_*L*_ < 1) or irreversible (*R*_*L*_ = 0) [41]. Furthermore, the biosorption behavior of the Cr ions on the CLB biomass was also described by the Langmuir type equation related to surface coverage (*θ*), which is defined as the fraction of the adsorption sites occupied by the solute in the equilibrium and expressed as follows:

$$\theta =\frac{b\;C_{0}}{1+b\;C_{0}}$$ (11)

The Freundlich model is an empirical equation that applies to non-ideal adsorption equilibrium on heterogeneous surfaces and also to multi-layer adsorption, suggesting that binding sites are not equivalent and/or independent [42]. This model assumes that there is an infinite supply of unreacted adsorption sites, that the stronger binding sites are occupied first, that binding strength decreases with the increasing degree of site occupation, and that there is a logarithmic reduction of the affinity between solute and adsorbent during surface coverage [43–45]. The Freundlich isotherm expression is the following:

qe=kFCe1nF (12)

where *k*_*F*_ [(mg g^-1^)(mg L^-1^)^-1/nF^] is a constant indicative of the relative adsorption capacity of the adsorbent and *n* is the heterogeneity factor, and its reciprocal indicates the intensity of adsorption [22,46].

### Thermodynamic study

To describe the thermodynamic behavior of Cr adsorption onto CLB biomass, relevant parameters such as Arrhenius activation energy (*E*_*a*_), and the changes in activation enthalpy (ΔH*), entropy (ΔS*), and Gibbs free energy (ΔG*) were calculated.

The activation energy (*E*_*a*_) was estimated by the Arrhenius equation, which is given below:

$$k=Ae^{\frac{-E_{a}}{RT}}$$ (13)

where *E*_*a*_ is the activation energy (J mol^-1^), *k* is the adsorption rate constant, which was obtained from the biosorption kinetics modeling of total Cr at different temperatures, *A* is the pre-exponential factor or frequency factor, *R* is the molar constant of gases (8.314 J mol^-1^ K^-1^), and *T* is the absolute solution temperature (K).

Furthermore, the change in activation enthalpy (ΔH*) and entropy (ΔS*) were calculated using the transition state theory equation [47]:

$$k=\frac{R\;T}{N\;h}e^{\frac{\Delta S*}{R}}e^{\frac{-\Delta H*}{RT}}$$ (14)

where *T* is the absolute solution temperature (K), *N* is Avogadro´s number, *h* is Planck´s constant, *R* is the molar constant of gases, and *k* is the adsorption rate constant.

The change in Gibbs free energy of activation (ΔG*) was determined at each assayed temperature by appling the following formula based on thermodynamic functions:

ΔG*=ΔH*−TΔS* (15)

### Data analysis

The Cr(VI) and total Cr removal experiments conducted in this work were reproducible within 5% error at most, and mean values from three independent replicates are reported herein.

All kinetic, isotherm and thermodynamic parameters of the models were evaluated by non-linear regression analysis of the experimental data using the *Curve Fitting Toolbox* 1.2.1 software (The MathWorks, Inc.). The determination coefficients (*R* ^*2*^) and the root mean squared error or standard error (*RMSE*) of the estimate were used as a measure of the goodness-of-fit of the mathematical models. Values of *R* ^*2*^ close to 1.0 and small *RMSE* values indicate better curve fitting.

### Analytical techniques

Cr(VI) and total Cr concentrations were quantified by photocolorimetric methods using a Genesys™ 10 UV-Visible spectrophotometer (Thermo Electron Scientific Instruments Corporation), following the procedures outlined in the Hach Water Analysis Handbook [48].

Cr(VI) concentration in solution was measured at 540 nm by the 1,5-diphenylcarbohydrazide method, using a single dry powder formulation called ChromaVer3^TM^ Chromium Reagent. This reagent contains an acidic buffer combined with 1,5-diphenylcarbohydrazide, which turns purple when Cr(VI) is present. Color intensity was directly proportional to the amount of Cr(VI) present.

Total Cr concentration in the filtrates (essentially, the sum of the Cr(III) and Cr(VI)) was analyzed by the alkaline hypobromite oxidation method [48]. In this method, the Cr(III) present in the samples is oxidized to Cr(VI) by the hypobromite ion at boiling temperature under strong alkaline conditions. Then, the samples are acidified and the Cr(VI) concentration, which equals the total Cr concentration, is determined by the 1,5-diphenylcarbohydrazide method. Cr(III) concentration in solution was estimated by subtracting residual Cr(VI) concentration from residual total Cr concentration [48].

Cr(VI) and total Cr concentrations were proportional to their optical absorbance and quantified by external standards, with a ten-point calibration curve.

## Results and Discussion

### Characterization of CLB

**Fig 1:**
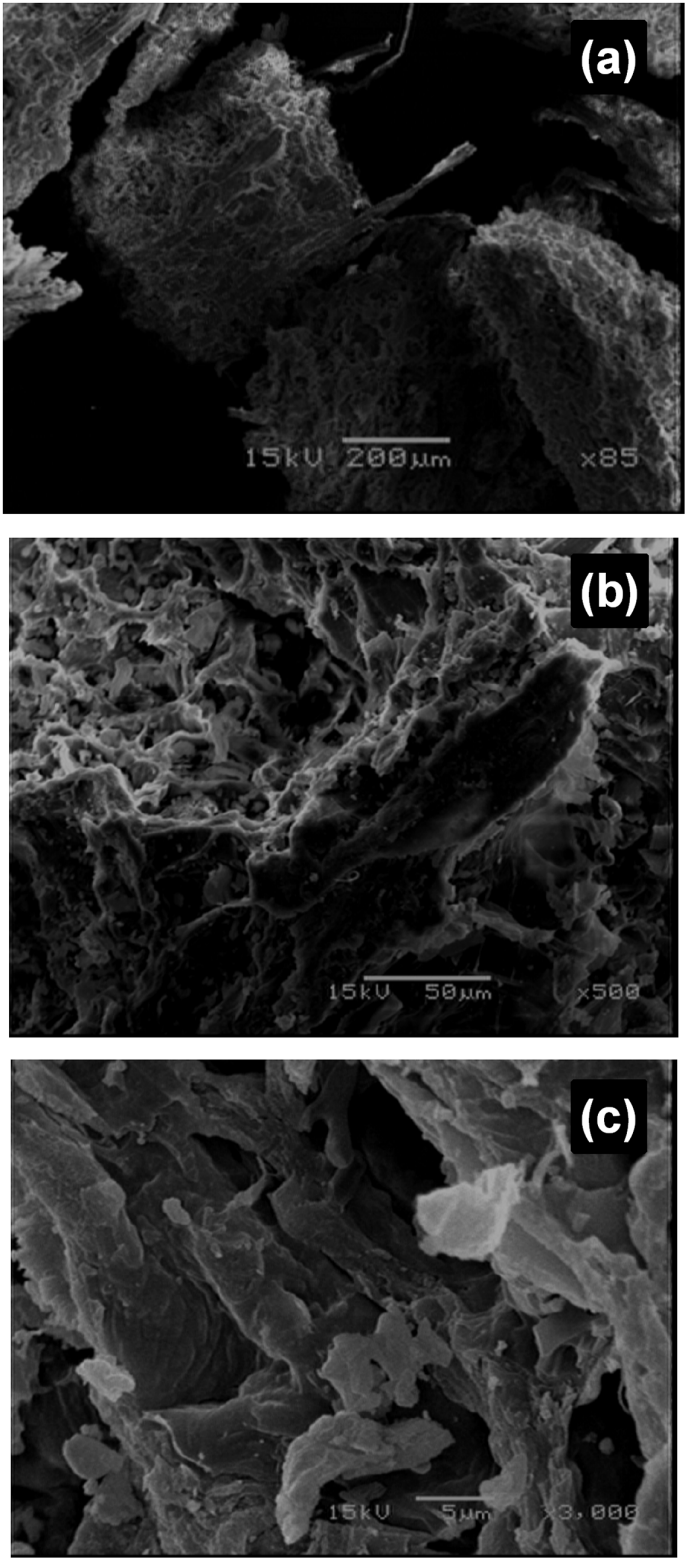
Scanning electron micrographs of CLB at (a) 85×, (b) 500× and (c) 3000× magnification.

Scanning electron microscopy (SEM) is widely used to study the morphological features and surface characteristics of biosorbent materials [49]. In the present study, SEM micrographs reveal that CLB has a rough and porous surface texture, with irregular pores that have a diameter exceeding 50 nm, which indicates that CLB has a macroporous structure (Fig 1). In addition, CLB has an irregular structure, thus making it possible for the biosorption of chromium ions on different parts of the biosorbent.

The bulk density, swollen particle density and floatability of CLB were found to be 239 kg m^-3^, 1067 kg m^-3^ and 24.7%, respectively.

### Effect of initial Cr(VI) concentration on Cr(VI) and total Cr removal

**Fig 2:**
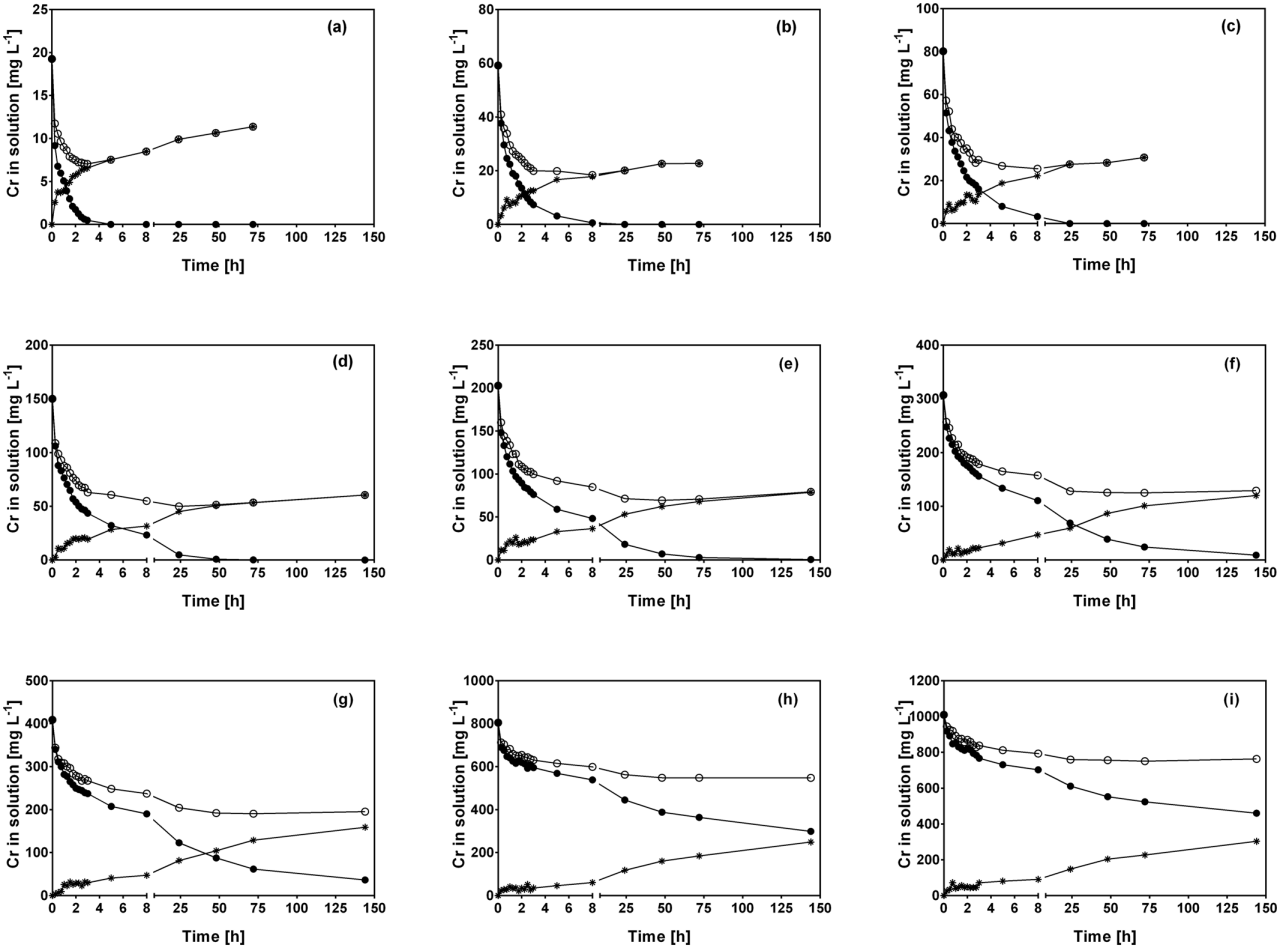
Variations of Cr(VI) (●), total Cr (○) and Cr(III) (✳) concentration with respect to contact time at different initial Cr(VI) concentrations. [Concentration (mg L^-1^): (a) 20, (b) 60, (c) 80, (d) 150, (e) 200, (f) 300, (g) 400, (h) 800, (i) 1000]. CLB concentration: 1 g L^-1^; pH = 1.5±0.1; temperature = 28±2°C.

Fig 2 displays the kinetic profiles of Cr(VI) and total Cr concentration obtained when CLB was mixed with Cr(VI) solutions at various initial Cr(VI) concentrations assayed in the present work (data obtained for other initial Cr(VI) concentrations tested are not shown).

CLB was able to remove the Cr(VI) completely at initial Cr(VI) concentrations of up to 250 mg L^-1^; however, total Cr (Cr(III) and Cr(VI)) was not entirely removed at any initial Cr(VI) concentration.

Residual total Cr concentration was higher than residual Cr(VI) concentration at all initial Cr(VI) concentrations and contact times assayed. This was due to the appearance of Cr(III) in the aqueous solution (Fig 2), which was not present at the start of the experiment, but was formed as a product of Cr(VI) reduction by organic compounds contained in CLB [27].

In experiments conducted at initial Cr(VI) concentrations ranging from 10 to 80 mg L^-1^, residual total Cr concentration reached its lowest level at a similar contact time as that required for complete Cr(VI) removal (Fig 2a–2c), which suggests that total Cr removal by CLB is closely related to the presence of Cr(VI) in solution. In contrast, at higher initial Cr(VI) concentrations (150–1000 mg L^-1^), residual Cr(VI) was still present in the solution when total Cr reached its lowest level (Fig 2d–2i). At these initial Cr(VI) concentrations, lowest total Cr levels were reached at 24 or 48 h of contact and remained constant thereafter; in contrast, Cr(VI) and Cr(III) in solution continued changing after 24 h. The above results indicate that the time required to reach the equilibrium of residual total Cr concentration clearly depended on the initial Cr(VI) concentration.

**Fig 3:**
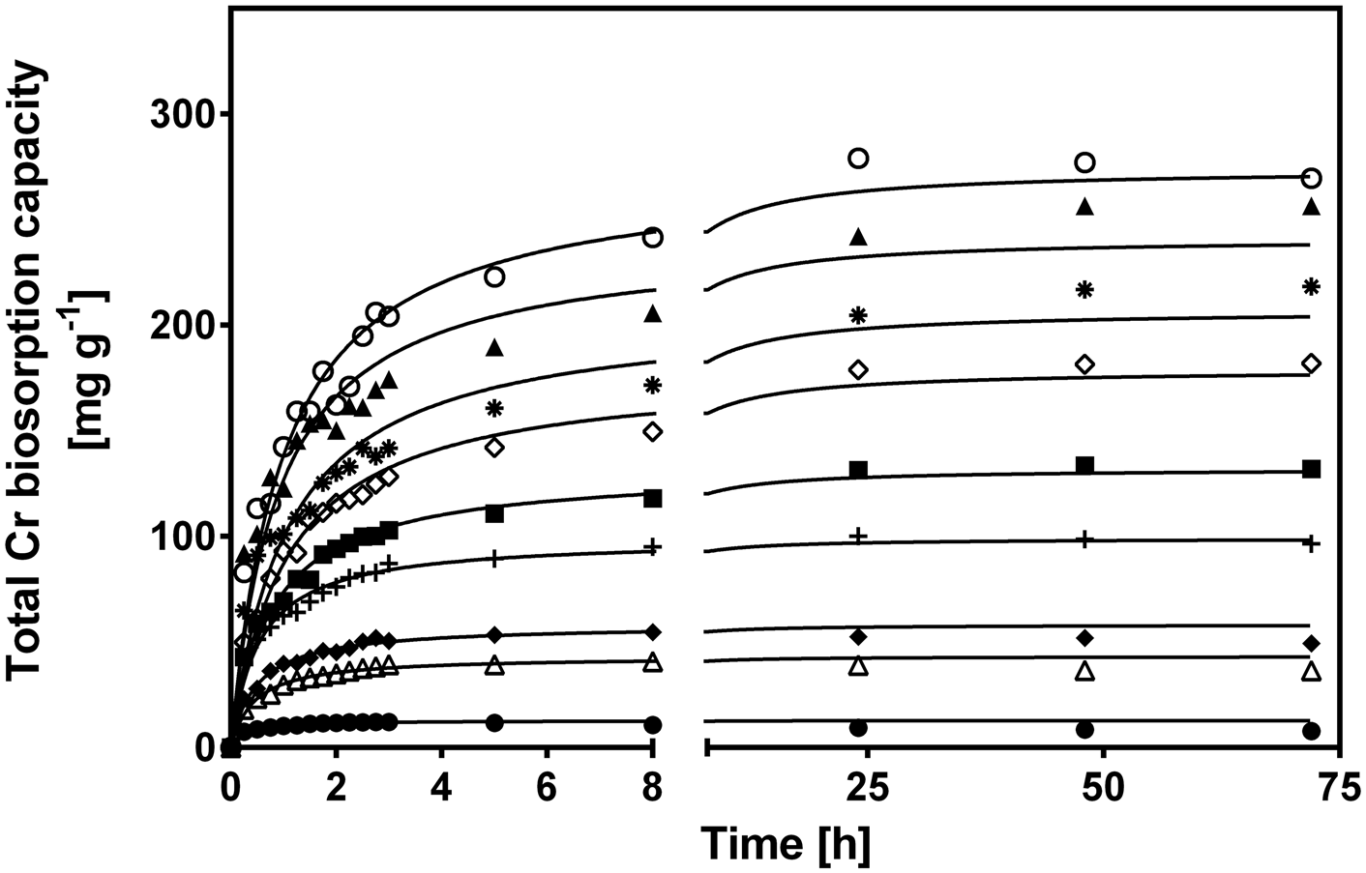
Effect of initial Cr(VI) concentration on total Cr biosorption capacity of CLB. [Initial Cr(VI) concentration (mg L^-1^): (●) 20, (△) 60, (◆) 100, (+) 150, (■) 200, (◇) 250, (✳) 400, (▲) 800, (○) 1000;—pseudo-second-order model prediction]. CLB concentration: 1 g L^-1^; pH = 1.5±0.1; temperature = 28±2°C.

Fig 3 shows the change in CLB total Cr biosorption capacity with respect to biosorption time for various initial Cr(VI) concentrations. The biosorption curves were single, smooth and continuous leading to saturation of Cr binding sites at the CLB surface. Regardless of initial Cr(VI) concentration, the total Cr biosorption capacity of CLB gradually rose as contact time elapsed, until it reached a maximum and constant value, which corresponds to the biosorption capacity at equilibrium (*q*_*exp*_). This value (*q*_*exp*_) rose from 7.08 to 275 mg g^-1^ as initial Cr(VI) concentration increased from 10 to 1000 mg L^-1^ (Table 1).

**Table 1:** Kinetic parameters of the Elovich, fractional power, pseudo-first-order and pseudo-second-order models for total Cr biosorption onto *Cupressus lusitanica* bark at different initial Cr(VI) concentrations.

**[Cr(VI)] (mg L** ^**-1**^ **)**		**Elovich**				**Fractional power**				
		α (mg g^-1^ h^-1^)	β (g mg^-1^)	*R* ^*2*^	*RMSE*	*k* (mg g^-1^)	*v* (h^-1^)	*kv* (mg g^-1^ h^-1^)	*R* ^*2*^	*RMSE*
10		669	1.08	0.880	0.289	6.06	0.143	0.866	0.846	0.832
20		617	5.74E-1	0.903	0.485	10.1	0.159	1.62	0.864	0.572
40		453	2.25E-1	0.932	1.13	20.5	0.186	3.82	0.875	1.53
60		425	1.39E-1	0.942	1.20	29.3	0.209	6.10	0.884	2.37
80		447	9.87E-2	0.945	2.29	38.2	0.222	8.50	0.887	3.29
100		1026	9.87E-2	0.898	3.94	47.3	0.165	7.79	0.822	5.20
150		1235	6.92E-2	0.933	4.48	64.9	0.171	11.1	0.862	6.42
200		1726	6.07E-2	0.913	8.08	78.8	0.147	11.6	0.836	11.1
250		1316	4.87E-2	0.933	8.74	88.2	0.160	14.1	0.858	12.7
300		1052	4.03E-2	0.955	8.52	96.2	0.173	16.7	0.883	13.7
350		1359	3.94E-2	0.972	6.76	104	0.169	17.6	0.920	11.6
400		1266	3.51E-2	0.984	5.58	111	0.177	19.6	0.943	10.8
600		1512	3.14E-2	0.969	9.38	126	0.173	21.8	0.914	15.5
800		2230	3.17E-2	0.982	6.83	137	0.164	22.5	0.946	11.8
1000		2089	2.74E-2	0.924	16.6	152	0.164	24.9	0.851	23.2
**[Cr(VI)] (mg L** ^**-1**^ **)**	***q***_***exp***_ **(mg g** ^**-1**^ **)**	**Pseudo-first-order**				**Pseudo-second-order**				
		*q*_*e1*_ (mg g^-1^)	*k*_*1*_ (h^-1^)	*R* ^*2*^	*RMSE*	*q*_*e2*_ (mg g^-1^)	*h* (mg g^-1^ h^-1^)	*k*_*2*_ (g mg^-1^ h^-1^)	*R* ^*2*^	*RMSE*
10	7.08	6.81	3.42	0.962	0.383	7.41	41.7	7.59E-1	0.987	0.223
20	12.0	10.9	3.86	0.816	1.29	12.8	61.0	3.72E-1	0.989	0.353
40	27.1	25.6	1.96	0.962	1.43	28.7	82.2	1.00E-1	0.993	0.632
60	40.0	38.0	1.66	0.962	2.16	43.1	101	5.45E-2	0.991	1.08
80	53.0	50.8	1.54	0.962	2.90	58.1	122	3.62E-2	0.990	1.51
100	69.7	64.1	1.28	0.917	5.26	71.3	138	2.72E-2	0.975	2.91
150	97.6	88.5	1.28	0.918	7.26	99.0	187	1.90E-2	0.979	3.68
200	132	121	0.822	0.923	9.70	132	167	9.62E-3	0.985	4.33
250	157	144	0.724	0.917	12.0	156	170	7.01E-3	0.981	5.73
300	181	165	0.651	0.915	14.1	179	170	5.29E-3	0.982	6.54
350	195	173	0.696	0.856	19.1	188	192	5.45E-3	0.975	6.1
400	218	192	0.626	0.850	21.4	207	193	4.49E-3	0.985	5.7
600	246	218	0.660	0.897	20.6	234	232	4.23E-3	0.968	11.6
800	256	222	0.735	0.821	27.0	241	268	4.62E-3	0.985	6.3
1000	275	254	0.675	0.906	22.7	274	280	3.73E-3	0.971	12.6

The rise in equilibrium biosorption capacity may be due to greater availability of Cr ions in the biosorption solution. Higher initial Cr(VI) concentration also increases the Cr concentration gradient, which provides a stronger thermodynamic driving force to overcome the mass transfer resistance of Cr ions from the aqueous to the solid phase; this increases the probability of collision between ions and active CLB sites, thus leading to enhanced biosorption capacity [11,50]. The saturation degree of the CLB surface clearly depended on initial Cr(VI) concentration.

Rising initial Cr(VI) concentration within the 10 to 150 mg L^-1^ range, increased the time needed to reach equilibrium; e.g., at 10, 20, 40, 60 and 150 mg L^-1^, the time was 2.5, 3, 5, 8 and 24 h, respectively. In contrast, at initial Cr(VI) higher than 150 mg L^-1^, equilibrium was reached after approximately 48 h.

The above results clearly show that Cr(VI) and total Cr removal from aqueous solutions by CLB strongly depended on initial Cr(VI) concentration.

### Effect of temperature on the kinetics of Cr(VI) and total Cr removal

**Fig 4:**
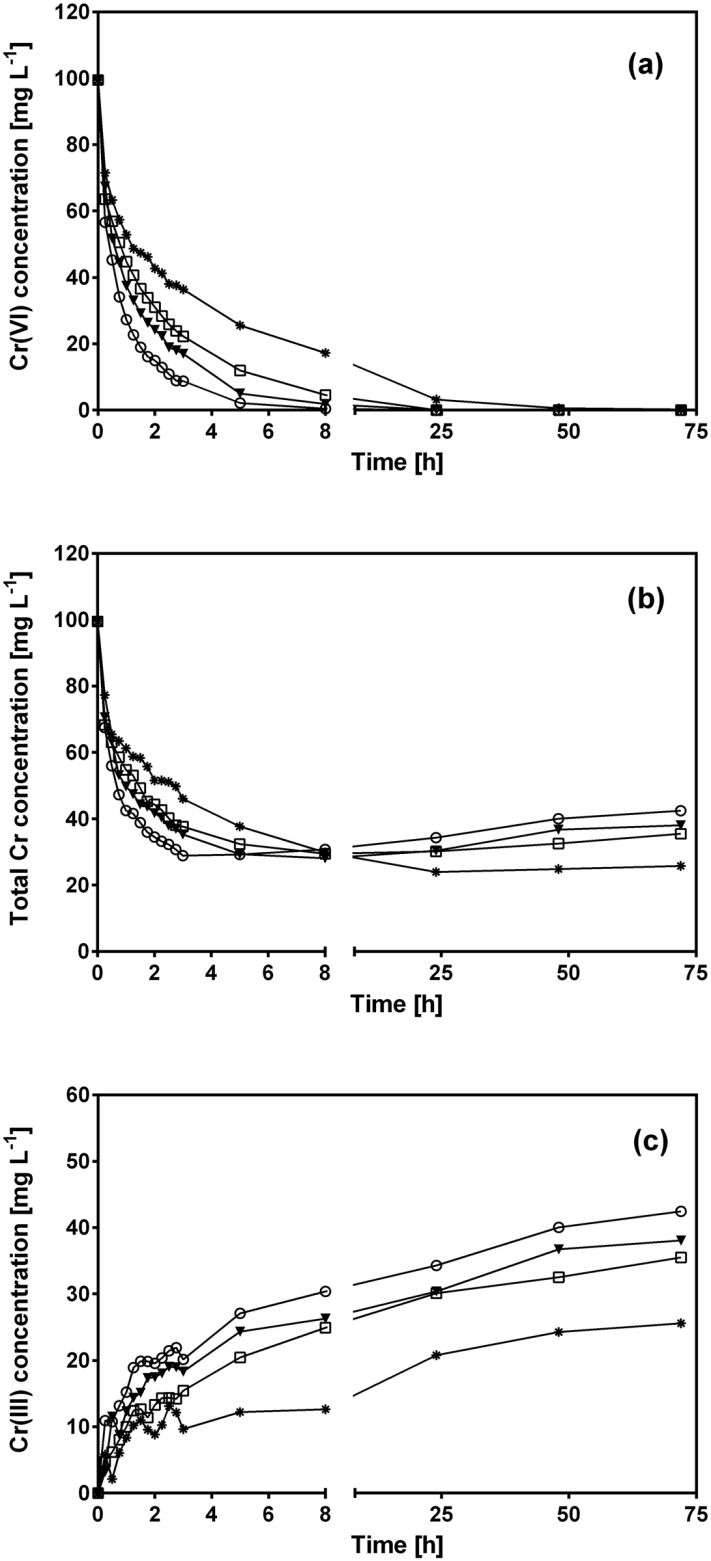
Variations of Cr(VI) (a), total Cr (b) and Cr(III) (c) concentration with respect to contact time at different solution temperatures. [Temperature (°C): (✳) 15, (⬜) 28, (▼) 35, (○) 45]. CLB concentration: 1 g L^-1^; pH = 1.5±0.1; initial Cr(VI) concentration = 100 mg L^-1^.

Fig 4 displays variation of the Cr(VI), total Cr and Cr(III) concentration profiles as a function of contact time with CLB at temperatures in the range from 15 to 45°C.

As temperature increased, residual Cr(VI) decreased more quickly (Fig 4a) and, consequently, Cr(VI) was entirely removed from the aqueous solution at shorter contact times, e.g. 72, 24, 24 and 8 h of contact time were required to remove all initial Cr(VI) at 15, 28, 35 and 45°C, respectively.

Fig 4b shows that temperature also affected total Cr removal by CLB, but to a lesser extent than for Cr(VI). CLB removed all initial Cr(VI) at temperatures from 15 to 45°C, but was not able to remove the entire total Cr. Residual total Cr concentration was considerably higher at all temperatures and contact times than residual Cr(VI) concentration. As mentioned before, this was due to Cr(VI) which was reduced to Cr(III) by CLB and released into the aqueous solution (Fig 4c). The highest Cr(III) levels were produced at 45°C, and decreased as temperature fell (Fig 4c). Thus, reduction of Cr(VI) to Cr(III) by CLB was favored at high temperatures.

At temperatures from 28 to 45°C, residual total Cr concentration reached its lowest level at a similar contact time as required for complete Cr(VI) removal. As contact time elapsed, total Cr levels began to increase slightly even when no more Cr(VI) was present in the aqueous solution (Fig 4a and 4b), which indicates that part of the Cr biosorbed by CLB was released (desorbed) into the solution as Cr(III). These results indicate that as temperature increased from 28 to 45°C, desorbed Cr(III) levels increased.

**Fig 5:**
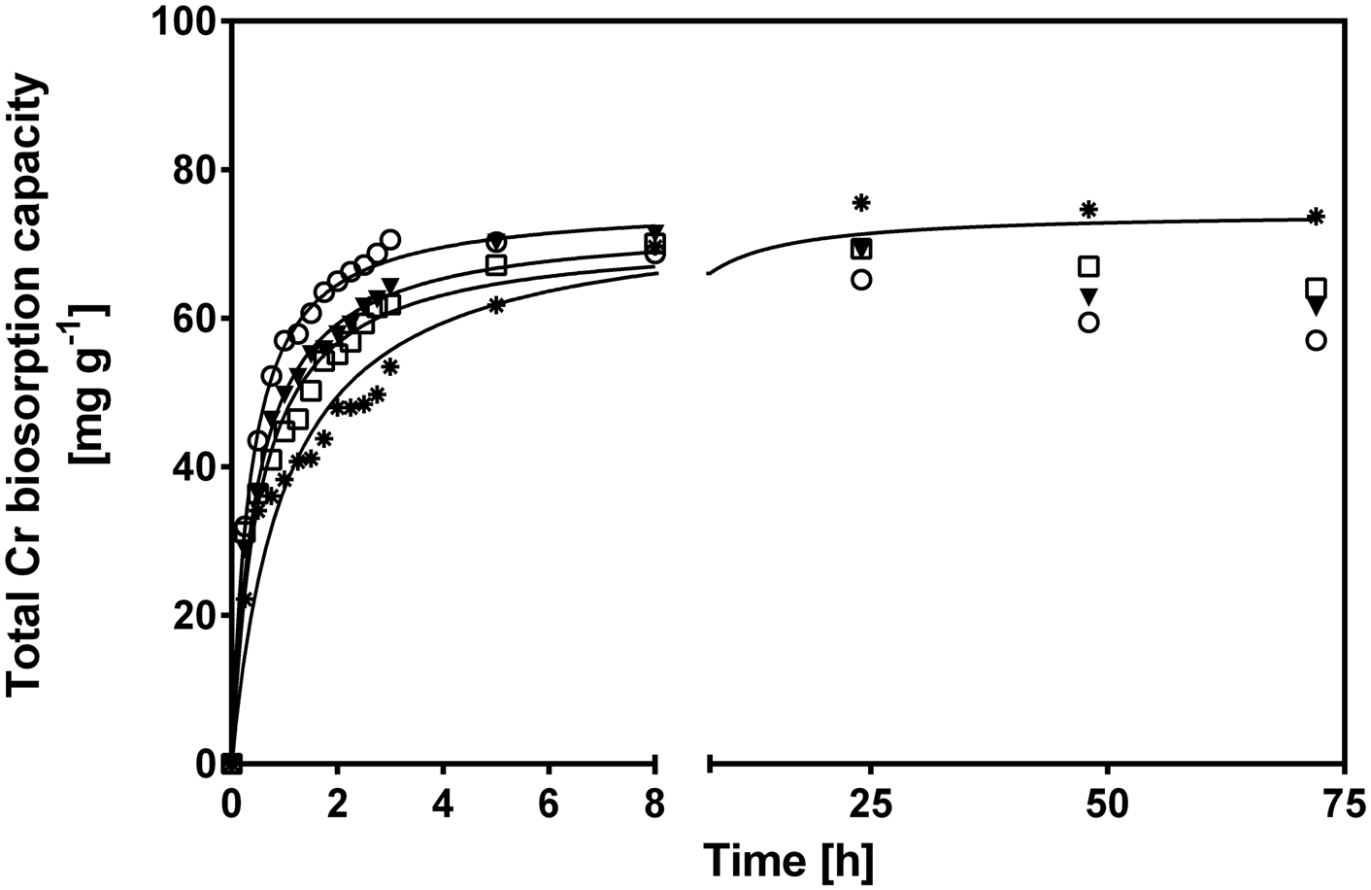
Influence of temperature on total Cr biosorption capacity of CLB. [Temperature (°C): (✳) 15, (⬜) 28, (▼) 35, (○) 45; — pseudo-second-order model prediction]. CLB concentration: 1 g L^-1^; pH = 1.5±0.1; initial Cr(VI) concentration = 100 mg L^-1^.

Fig 5 shows total Cr biosorption capacity of CLB as a function of contact time for the assayed temperatures (15–45°C). During the first hours of experimentation, the total Cr biosorption rate increased as temperature rose. These results indicate the endothermic nature of total Cr biosorption onto CLB. The observed increase may be due to several factors: a rise in kinetic energy which facilitates the access of Cr ions to active CLB biosorption sites; a decrease in the thickness of the CLB boundary layer, which in turn decreases the mass transfer resistance enabling Cr ion diffusion into the CLB surface; an increase in CLB surface activity, which results in higher affinity for Cr ions, and/or an increase in the active biosorption sites [11,50].

Previous diffuse reflectance infrared Fourier transform spectroscopy (DRIFTS) studies showed the presence of absorption bands corresponding to aromatic compounds such as lignin and tannins, cellulose molecules, O-H groups, alkyl radicals and other saturated aliphatic groups, as well as carboxyl groups on the surface of CLB [27]. Based on the DRIFTS results, it was proposed that the mechanism of total chromium biosorption from Cr(VI) solutions onto CLB involves four reaction steps: (1) formation of Cr(VI) complexes resulting from the interaction between Cr(VI) ions and oxygen-containing groups (adsorption of Cr(VI) oxyanions), (2) Cr(VI) to Cr(III) reduction, (3) carboxyl group formation resulting from oxidation of oxygen-containing groups, and (4) Cr(III) interaction with carboxyl groups to form Cr(III)-carboxylate complexes [27]. It is therefore reasonable to assume that each reaction step will be differently affected by temperature and, consequently, temperature will affect the overall total Cr biosorption performance.

On the other hand, total Cr biosorption capacity at 8 h of contact time was similar at all assayed temperatures (Fig 5), ranging between 68.7 and 71.4 mg g^-1^. At longer contact times, the biosorption capacity slightly increased reaching an equilibrium adsorption capacity of 74.6 mg g^-1^ at 15°C; in contrast, at higher temperatures, the biosorption capacity decreased to 64, 61 and 57 mg g^-1^ at 28, 35 and 45°C, respectively, which was due to the desorption of Cr ions from CLB (Fig 5).

It is evident from the above results that temperature affected the biosorption rate of Cr ions, Cr(VI) reduction to Cr(III) and the extent of Cr(III) desorption.

### Kinetic modeling of the total Cr biosorption process

The Elovich, fractional power, pseudo-first-order and pseudo-second-order models were used in the present work to model the kinetic process of total Cr biosorption onto CLB at different initial Cr(VI) concentrations and temperatures. The data obtained during Cr desorption from CLB at temperatures ranging from 28 to 45°C were not considered for the modeling of the total Cr biosorption kinetics at different temperatures.

Tables 1 and 2 show the experimental equilibrium biosorption capacity (*q*_*exp*_), the kinetic parameter values of the Elovich, fractional power, pseudo-first-order and pseudo-second-order models for total Cr biosorption by CLB at initial Cr(VI) concentrations from 10 to 1000 mg L^-1^ and temperatures from 15 to 45°C, along with the corresponding *R* ^*2*^ and *RMSE* values.

**Table 2:** Kinetic parameters of the Elovich, fractional power, pseudo-first-order and pseudo-second-order models for total Cr biosorption onto *Cupressus lusitanica* bark at different temperatures. Initial Cr(VI) concentration: 100 mg L^-1^.

**Temperature (°C)**		**Elovich**				**Fractional power**				
		α (mg g^-1^ h^-1^)	β (g mg^-1^)	*R* ^*2*^	*RMSE*	*k* (mg g^-1^)	*v* (h^-1^)	*kv* (mg g^-1^ h^-1^)	*R* ^*2*^	*RMSE*
15		562	1.01E-1	0.933	4.19	41.06	0.1653	6.79	0.869	5.85
28		1026	9.87E-2	0.898	3.94	39.3	0.231	9.08	0.940	3.50
35		1335	9.95E-2	0.856	4.75	49.9	0.153	7.63	0.763	6.09
45		1272	8.63E-2	0.873	4.15	54.5	0.179	9.76	0.798	5.24
**Temperature (°C)**	***q***_***exp***_ **(mg g** ^**-1**^ **)**	**Pseudo-first-order**				**Pseudo-second-order**				
		*q*_*e1*_ (mg g^-1^)	*k*_*1*_ (h^-1^)	*R* ^*2*^	*RMSE*	*q*_*e2*_ (mg g^-1^)	*h* (mg g^-1^ h^-1^)	*k*_*2*_ (g mg^-1^ h^-1^)	*R* ^*2*^	*RMSE*
15	74.6	69.6	0.634	0.876	7.02	74.3	74.1	1.34E-2	0.955	4.02
28	69.7	64.1	1.28	0.917	0.911	71.3	138	2.72E-2	0.975	0.91
35	70.2	65.2	1.48	0.952	0.948	72.9	158	2.98E-2	0.990	0.87
45	69.9	67.5	2.00	0.983	2.6	75.5	222	3.89E-2	0.994	1.54

The pseudo-second-order model clearly yielded the highest *R* ^*2*^ and the lowest *RMSE* values of the four assayed kinetic models at the initial Cr(VI) concentrations and temperatures assayed, which indicates that the experimental data are in good agreement with the pseudo-second-order model. Furthermore, the pseudo-second-order model successfully described the variations in total Cr biosorption capacity at the different initial Cr(VI) concentrations and temperatures assayed (continuous lines in Figs 3 and 5), and predicted values of biosorption capacity at equilibrium very close to the equilibrium capacities obtained experimentally (Tables 1 and 2). Incidentally, the pseudo-second-order model had been previously found to be the most suitable model to describe the kinetic profiles of total Cr biosorption by CLB at different solution pH levels [27]. The fitness of total Cr biosorption kinetics to the pseudo-second-order model suggests that the rate-limiting step in biosorption of Cr ions onto CLB is probably a chemical sorption (chemisorption) involving valence forces through the sharing or exchange of electrons between the CLB surface and Cr ions [38]. This model assumes that two reactions are occurring, the first one is fast and reaches equilibrium quickly and the second is a slower reaction that can continue for long time periods. The reactions can occur either in series or in parallel [51]. The pseudo-second-order model also describes the experimental kinetic data of total Cr biosorption by different biosorbents [28,52–55].

For the pseudo-second-order model, with increasing initial Cr(VI) concentration the initial sorption rate (*h*) also tended to rise (Table 1), while the rate constant (*k*_*2*_) gradually decreased until it reached a minimum, almost constant value of about 10^−3^ g mg^-1^ h^-1^, which occurred at initial Cr(VI) concentrations higher than 150 mg L^-1^. The trend shown by *k*_*2*_ values agrees with the experimental data since at initial Cr(VI) concentrations lower than 150 mg L^-1^, the time required to reach the equilibrium biosorption capacity was lower and therefore the *k*_*2*_ values were higher; whereas at higher initial Cr(VI) concentrations, the contact time needed to reach equilibrium was similar and this was reflected in the almost constant values of *k*_2_ [11,50,56]. A qualitatively similar behavior for the *h* and *k*_*2*_ parameters of the pseudo-second-order model was observed in a dynamics study on the effect of initial Cr(VI) concentration on Cr biosorption onto waste acorn of *Quercus ithaburensis* [57].

Results also showed that the *h* and *k*_*2*_ values increased with a rise in temperature of the biosorption system (Table 2). This behavior may be explained by an increase in the interactions between Cr ions and CLB with rising temperature, and confirms a faster Cr biosorption rate at higher temperatures, as well as the endothermic nature of the biosorption process [58].

### Biosorption isotherm study

**Fig 6:**
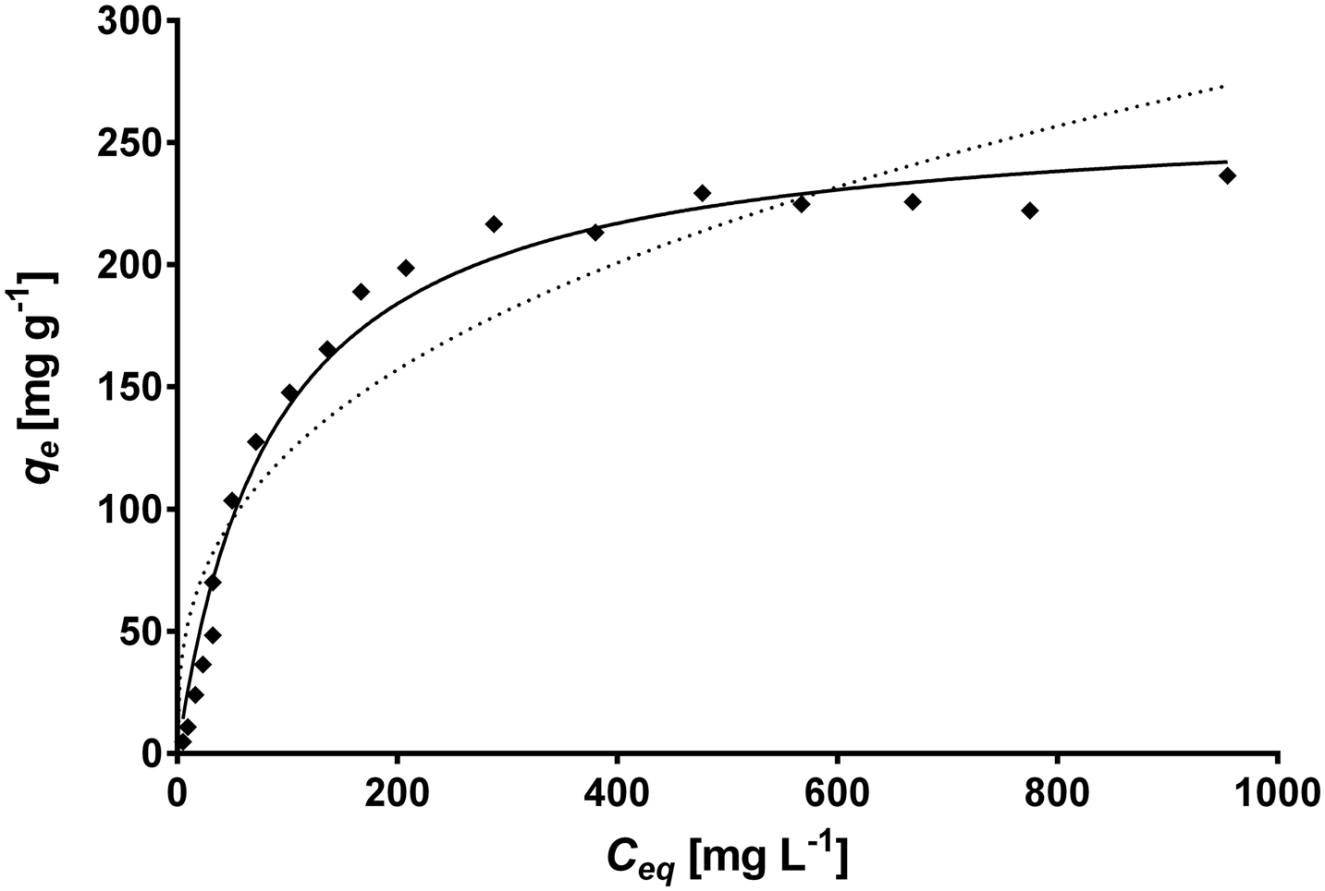
Comparison between the experimental isotherm data (◆) and the predicted isotherm data derived from the Langmuir (—) and Freundlich (---) models for total Cr biosorption onto CLB. Conditions: CLB concentration: 1 g L^-1^; pH = 1.5±0.1; temperature = 28±2°C.

The equilibrium biosorption isotherm is an important parameter that describes the interactions of the sorbate (e.g. metal ions) with the biosorbent and is therefore required to understand the mechanism of the biosorption. Fig 6 displays the experimental biosorption isotherm of total Cr at pH 1.5 and 28°C. The equilibrium biosorption capacity steadily rises with the increase in equilibrium total Cr concentration (*C*_*eq*_) up to about 585 mg L^-1^, after which it reaches a saturation value. The concave shape of the total Cr biosorption isotherm resembles the type L isotherm of the Giles classification [59], which is generally associated with the sorption of a solute monolayer with minimum competition for the solvent. Furthermore, this type of isotherm indicates that the available sorption sites gradually decrease as the solute concentration in solution increases, i.e. it suggests a progressive saturation of the biosorbent. The L-type isotherm also suggests high affinity between CLB and Cr ions [40,50,60].

The Langmuir and Freundlich isotherm models, which are widely used for modeling adsorption isotherms, were used in this work to describe the experimental equilibrium data of total Cr biosorption onto CLB. The comparative analysis of *R* ^*2*^ and *RMSE* values shown in Table 3 indicate that the biosorption pattern for total Cr on CLB is best described by the Langmuir model (*R* ^*2*^ = 0.990; *RMSE* = 10.16), rather than by the Freundlich model (*R* ^*2*^ = 0.892; *RMSE* = 32.7). Moreover, the predicted equilibrium biosorption capacity obtained with the Langmuir model (continuous line in Fig 6) matched experimental value closely. These results are in accordance with the shape of the experimental biosorption isotherm, which exhibits Langmuirian behavior. The fact that the Langmuir model fits the experimental data very well may be due to a homogeneous distribution of active sites on the CLB biomass, since the Langmuir model assumes that the surface is homogeneous. This model also assumes the formation of a uniform monolayer, a finite number of adsorption sites, and no interaction between adsorbate ions adsorbed on neighboring sites. The good fitness of the Langmuir model to the experimental equilibrium biosorption data also suggests that Cr ions are possibly biosorbed onto CLB by chemisorption because chemical adsorption involves a monolayer coverage, rather than a multilayer adsorption as in the case of physical adsorption [61,62]. Thus, results from the kinetic and the equilibrium modeling indicate that total Cr biosorption by CLB occurs by chemisorption.

**Table 3:** Parameters of the Langmuir and Freundlich isotherm models for total Cr biosorption onto *C*. *lusitanica* bark.

**Langmuir**	
*Q*_*0*_ (mg g^-1)^	305.4
*b* (L mg^-1^)	0.0102
*R* ^*2*^	0.990
*RMSE*	10.16
**Freundlich**	
*k*_*F*_ [(mg g^-1^)(mg L^-1^)^-1/nF^]	24.1
*n*_*F*_	2.69
*R* ^*2*^	0.892
*RMSE*	32.7

The Langmuir model has been widely used to estimate the maximum biosorption capacity whenever it was not possible to reach it experimentally, and contains the two most important parameters in a biosorption process, *Q*_*o*_ and *b*. The first (*Q*_*o*_) is attributed to the maximum capacity of biosorption to complete saturation of the biosorbent, and the second (*b*) is a constant related to the affinity between biosorbent and sorbate [58]. These two parameters have great practical importance for engineering design and scale-up of biosorption processes [60].

**Fig 7:**
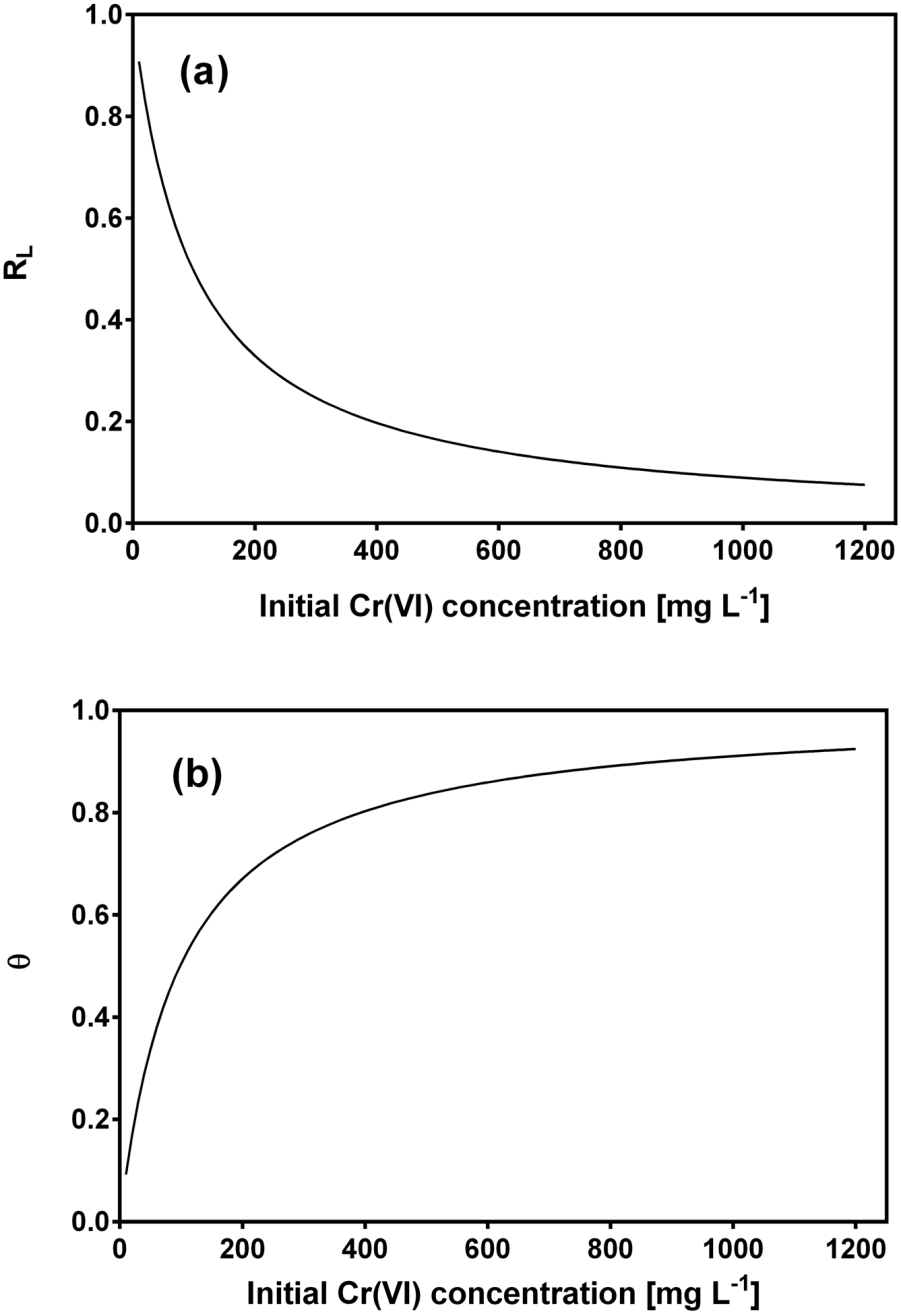
Dependence of separation factor (a) and surface coverage (b) on initial Cr(VI) concentration.

The Langmuir model predicted a saturated monolayer adsorption capacity (*Q*_*o*_) of 305.4 mg g^-1^, which adequately matched the experimental value of total Cr biosorption capacity at equilibrium (275 mg g^-1^), and a biosorption equilibrium constant (*b*) of 0.0102 L mg^-1^ within the interval of *b* values (0–1) which indicate that the biosorption of total Cr onto CLB is favorable under the present experimental conditions. To confirm the favorability of the total Cr biosorption process, the dimensionless separation factor was calculated and found to decrease from 0.908 to 0.0895 as initial Cr(VI) concentration increased from 10 to 1200 mg L^-1^ (Fig 7a), which confirms that biosorption of total Cr onto CLB increases as the initial Cr(VI) concentration rises. Moreover, the *R*_*L*_ values were between 0 and 1, which indicates favorable biosorption of total Cr onto CLB at all the metal concentrations assayed [60,63]. The surface coverage (*θ*) values approached unity with increasing initial Cr(VI) concentration (Fig 7b), which indicates that the CLB surface was nearly fully covered with a monomolecular layer at the higher metal concentrations. The effectiveness of CLB for the biosorption of total Cr is thus confirmed. It was also apparent that the surface coverage ceases to vary significantly at high concentrations of Cr(VI) and the reaction rate becomes almost independent of the metal concentration [63].

A comparison of the maximum total Cr biosorption capacity of CLB with reports for different biosorbents is summarized in Table 4 [14,24,25,62,64–80]. The total Cr biosorption capacity of CLB is similar to that reported for *Prunus domestica* L. bark [78], lower than that obtained for the dendrimer poly(amidoamine)-grafted cellulose nanofibril aerogels [80], and significantly higher than for most other adsorbents and biosorbents reported in the literature. Thus, CLB is one the best adsorbents currently available for the biosorption of total chromium from aqueous solutions, and is therefore an effective, renewable and promising material for remediation of Cr(VI)-contaminated water and wastewater.

**Table 4:** Comparison of maximum total Cr adsorption capacity predicted by the Langmuir model for various adsorbents.

Material	*Q*_*0*_ (mg g^-1^)	pH	Temperature (°C)	Reference
*Chlamydomonas reinhardtii*	24.9	2.0	25	[64]
*Sargassum siliquosum*	66.4	2.1	30	[65]
*Sargassum muticum*	196.1	2.0	20	[66]
*Sargassum muticum*	185.2	2.0	50	[66]
*Nannochloris oculata* biomass after lipid extraction	37.7	2.0	-	[67]
*Fucus vesiculosus*	42.62	2.0	-	[68]
*Fucus spiralis*	35.35	2.0	-	[68]
*Ulva lactuca*	27.55	2.0	-	[68]
*Ulva* spp.	30.15	2.0	-	[68]
*Palmaria palmata*	33.79	2.0	-	[68]
*Polysiphonia lanosa*	45.75	2.0	-	[68]
*Ceramium virgatum*	26.5	1.5	20	[69]
*Oedogonium hatei* (raw)	31.0	2.0	45	[70]
*Oedogonium hatei* (acid treated)	35.2	2.0	45	[70]
*Penicillium chrysogenum* (raw)	9.11	4.6	25	[71]
*Penicillium chrysogenum* (polyethylenimine-modified)	279.2	4.6	25	[71]
*Grape stalk*	59.8	3.0	25	[72]
*Olive stone*	9.0	2.0	25	[72]
*Yohimbe bark*	42.5	2.0	25	[72]
Cork	17.0	3.0	25	[72]
Chemically-modified hazelnut shell	17.7	2.0	20	[73]
Na_2_HPO_4_-pretreated pine sawdust	121.95	2.0	40	[62]
Lignocellulosic substrate extracted from wheat bran	37.43	2.1	-	[25]
Brazilian-pine fruit wastes (*Araucaria angustifolia*)	240	2.0	25	[14]
Crab (*Ucides cordatus*) shell	28.07	2.0	-	[74]
Persimmon tannin gel	274	3.0	30	[75]
Iron(III) hydroxide-loaded sugar beet pulp	5.12	4.4	25	[76]
Commercial activated carbon	7.61	-	30	[77]
HCl-pretreated *Prunus domestica* L. bark	302.9	2.0	25	[78]
*Moringa aptera* pods	3.19	-	20	[79]
*Moringa aptera* pods	4.87	-	40	[79]
*Moringa aptera* pods	5.50	-	70	[79]
Dendrimer poly(amidoamine)-grafted cellulose nanofibril aerogel	377.36	2.0	-	[80]
*Aspergillus niger* MSR2	71.9	2.0	27	[24]
*C*. *lusitanica* bark	305	1.5	28	This work

### Biosorption thermodynamics

To evaluate the thermodynamic behavior of total Cr biosorption onto CLB and to gain insight into the Cr biosorption mechanism, relevant thermodynamic parameters such as the Arrhenius activation energy (*E*_*a*_), as well as changes in activation enthalpy (ΔH*), activation entropy (ΔS*) and Gibbs free energy of activation (ΔG*) were calculated in the present work (Table 5). For this purpose, we used the rate constants of the pseudo-second-order model obtained in the kinetic study at different temperatures.

**Table 5:** Thermodynamic parameters for total Cr biosorption onto *C*. *lusitanica* bark.

*E*_*a*_(kJ mol^-1^)	*A*(s^-1^)	ΔH*(kJ mol^-1^)	ΔS*(kJ mol^-1^ K^-1^)	T (°C)	ΔG*(kJ mol^-1^)
				15	100.0
				28	103.6
23.26	72.8	21.0	-0.274	35	105.4
				45	108.2

The *E*_*a*_ value for total Cr biosorption onto CLB was 23.26 kJ mol^-1^ (Table 5). The magnitude of this thermodynamic parameter confirms that CLB biosorbs Cr from Cr(VI) aqueous solutions by a chemical sorption reaction (chemisorption), because the interval of *E*_*a*_ values for chemisorption processes ranges from 8.4 to 83.7 kJ mol^-1^, while for physical adsorption the *E*_*a*_ it is not higher than 4.2 kJ mol^-1^ [81].

The values for ΔH* and ΔS* were 21.0 kJ mol^-1^ and -0.274 kJ mol^-1^ K^-1^, respectively. The positive value of ΔH* confirms the endothermic nature of the total Cr biosorption process, while the negative value of ΔS* indicates a loss of degrees of freedom when the activated complex is formed [47]. The association, fixation or immobilization of Cr ions as a result of biosorption is attributed to a decrease in the degree of freedom of Cr ions which gives rise to a negative ΔS* [82]. Similarly, negative ΔS* values indicate that Cr ions are stable on the CLB surface and that no significant change occurs in the internal structure of CLB during the biosorption process [15,83]. Furthermore, at all assayed temperatures the values of ΔG* were positive (100.0–108.2 kJ mol^-1^), indicating that the total Cr biosorption process is not spontaneous and therefore requires some energy from an external source in order to occur [15]. The adsorption process of Cr onto raw rutin, rutin resin [84], lignin-based resin [85] and *Aspergillus niger* MSR2 [24] were also found to be endothermic. Likewise, the biosorption of Cr ions onto *A*. *niger* MSR2 is not spontaneous at 295 and 310 K [24].

## Conclusions

The potential of CLB to biosorb total Cr from Cr(VI) aqueous solutions was explored in the present work. The characteristics of the biosorption process were affected by variables such as initial Cr(VI) concentration, contact time and temperature. A second-order chemical reaction describes the biosorption of total Cr onto the CLB biomass. The non-linear isotherm analysis of equilibrium data showed that the total Cr biosorption pattern of CLB adequately fits the Langmuir model. The biosorption process was endothermic and non-spontaneous. Results suggest that CLB is an effective low-cost biosorbent with high biosorption capacity to remove Cr from aqueous solutions.

## References

[1] Zhitkovich A. Importance of chromium-DNA adducts in mutagenicity and toxicity of chromium(VI). Chem Res Toxicol. 2005; 18: 3–11. 15651842 10.1021/tx049774+

[2] Eisler R. Chromium hazards to fish, wildlife, and invertebrates: a synoptic review Laurel, MD: Fish and Wildlife Service Biological Report. 1986; 85 (1.6).

[3] Stasinakis AS, Thomaidis NS, Mamais D, Karivali M, Lekkas TD. Chromium species behavior in the activated sludge process. Chemosphere. 2003; 52: 1059–1067. 12781239 10.1016/S0045-6535(03)00309-6

[4] Samuel MS, Abigail MEA, Ramalingam C. Biosorption of Cr(VI) by *Ceratocystis paradoxa* MSR2 using isotherm modelling, kinetic study and optimization of batch parameters using response surface methodology. PLos ONE 2015; 10(3): e0118999 10.1371/journal.pone.0118999 25822726 PMC4379012

[5] Masaki Y, Hirajima T, Sasaki K, Okibe N. Bioreduction and immobilization of hexavalent chromium by the extremely acidophilic Fe(III)-reducing bacterium *Acidocella aromatic* strain PFBC. Extremophiles 2015; 19: 495–503. 10.1007/s00792-015-0733-6 25651881

[6] Morales-Barrera L, Guillén-Jiménez FdM, Ortiz-Moreno A, Villegas-Garrido TL, Sandoval-Cabrera A, Hernández-Rodríguez CH, et al Isolation, identification and characterization of a *Hypocrea tawa* strain with high Cr(VI) reduction potential. Biochem Eng J. 2008; 40: 284–292.

[7] Lin CJ. The chemical transformations of chromium in natural waters-a model study. Water Air Soil Pollut. 2002; 139: 137–158.

[8] Gaggelli E, Berti F, D´Amelio N, Gaggelli N, Valensin G, Bovalini L, et al Metabolic pathways of carcinogenic chromium. Environ Health Perspect. 2002; 110(5): 733–738. 12426122 10.1289/ehp.02110s5733 PMC1241235

[9] Rakhunde R, Deshpande L, Juneja HD. Chemical speciation of chromium in water: A review. Crit Rev Environ Sci Technol. 2012; 42(7): 776–810.

[10] Viamajala S, Peyton BM, Sani RK, Apel WA, Petersen JN. Toxic effects of chromium (VI) on anaerobic and aerobic growth of *Shewanella oneidensis* MR-1. Biotechnol Prog. 2004; 20: 87–95. 14763828 10.1021/bp034131q

[11] Puentes-Cárdenas IJ, Pedroza-Rodríguez AM, Navarrete-López M, Villegas-Garrido TL, Cristiani-Urbina E. Biosorption of trivalent chromium from aqueous solutions by *Pleurotus ostreatus* biomass. Environ Eng Manag J. 2012; 11: 1741–1752.

[12] Kumar R, Bishnoi NR, Garima Bishnoi K. Biosorption of chromium(VI) from aqueous solution and electroplating wastewater using fungal biomass. Chem Eng J. 2008; 135: 202–208.

[13] Deng L, Su Y, Su H, Wang X, Zhu X. Sorption and desorption of lead (II) from wastewater by green algae *Cladophora fascicularis* . J Hazard Mater. 2007; 143: 220–225. 17049733 10.1016/j.jhazmat.2006.09.009

[14] Vaghetti JCP, Lima EC, Royer B, Brasil JL, da Cunha BM, Simon NM, et al Application of Brazilian-pine fruit coat as biosorbent to removal of Cr(VI) from aqueous solution-kinetics and equilibrium study. Biochem Eng J. 2008; 42: 67–76. 10.1016/j.jhazmat.2007.11.101 18178307

[15] Doğan M, Abak H, Alkan M. Adsorption of methylene blue onto hazelnut shell: kinetics, mechanism and activation parameters. J Hazard Mater. 2009; 164(1): 172–181. 10.1016/j.jhazmat.2008.07.155 18809255

[16] Khezami L, Capart R. Removal of chromium(VI) from aqueous solution by activated carbons: Kinetic and equilibrium studies. J Hazard Mater. 2005; B123: 223–231. 10.1016/j.jhazmat.2005.04.012 15913888

[17] Bayramoğlu G, Arica MY. Adsorption of Cr(VI) onto PEI immobilized acrylate-based magnetic beads: isotherms, kinetics and thermodynamics study. Chem Eng J. 2008; 139: 20–28.

[18] Weng CH, Sharma YC, Chu SH. Adsorption of Cr(VI) from aqueous solution by spent activated clay. J Hazard Mater. 2008; 155: 65–75. 18162297 10.1016/j.jhazmat.2007.11.029

[19] Aranda-García E, Morales-Barrera L, Pineda-Camacho G, Cristiani-Urbina E. Effect of pH, ionic strength, and background electrolytes on Cr(VI) and total chromium removal by acorn shell of *Quercus crassipes* Humb. & Bonpl. Environ Monit Assess. 2014; 186: 6207–6221. 10.1007/s10661-014-3849-8 24880725

[20] Aravindhan R, Fathima A, Selvamurugan M, Rao JR, Balachandran UN. Adsorption, desorption, and kinetic study on Cr(III) removal from aqueous solution using *Bacillus subtilis* biomass. Clean Technol Environ Policy. 2012; 14: 727–735.

[21] Ma W, Meng F, Cheng Z, Xin G, Duan S. Synthesis of macroporous silica biomass nanocomposite based on XG/MgSiO_3_ for the removal of toxic ions. Bioresour Technol. 2015; 186: 356–359. 10.1016/j.biortech.2015.03.133 25862015

[22] Mohan D, Pittman CU Jr. Activated carbons and low cost adsorbents for remediation of tri- and hexavalent chromium from water. J Hazard Mater. 2006; B137: 762–811. 10.1016/j.jhazmat.2006.06.060 16904258

[23] Saha B, Orvig C. Biosorbents for hexavalent chromium elimination from industrial and municipal effluents. Coord Chem Rev. 2010; 254: 2959–2972.

[24] Samuel M, Abigail EA, Chidambaram R. Isotherm modelling, kinetic study and optimization of batch parameters using response surface methodology for effective removal of Cr(VI) using fungal biomass. PLoS ONE 2015; 10(3): e0116884 10.1371/journal.pone.0116884 25786227 PMC4364747

[25] Dupont L, Guillon E. Removal of hexavalent chromium with a lignocellulosic substrate extracted from wheat bran. Environ Sci Technol. 2003; 37: 4235–4241. 14524458 10.1021/es0342345

[26] Gardea-Torresdey JL, Tiemann KJ, Armendariz V, Bess-Oberto L, Chianelli RR, Rios J, et al Characterization of Cr(VI) binding and reduction to Cr(III) by the agricultural byproducts of *Avena monida* (Oat) biomass. J Hazard Mater. 2000; 80(1–3): 175–188. 11080577 10.1016/s0304-3894(00)00301-0

[27] Netzahuatl-Muñoz AR, Guillén-Jiménez FdM, Chávez-Gómez B, Villegas-Garrido TL, Cristiani-Urbina E. Kinetic study of the effect of pH on hexavalent and trivalent chromium removal from aqueous solution by *Cupressus* lusitanica bark. Water Air Soil Pollut. 2012; 223: 625–641.

[28] Netzahuatl-Muñoz AR, Morales-Barrera L, Cristiani-Urbina MdC, Cristiani-Urbina E. Hexavalent chromium reduction and chromium biosorption by Prunus serotina bark. Fresen Environ Bull. 2012; 21(7): 1793–1801.

[29] Park D, Lim SR, Yun YS, Park JM. Reliable evidences that the removal mechanism of hexavalent chromium by natural biomaterials is adsorption-coupled reduction. Chemosphere. 2007; 70: 298–305. 17644158 10.1016/j.chemosphere.2007.06.007

[30] Yang L, Chen JP. Biosorption of hexavalent chromium onto raw and chemically modified *Sargassum* sp. Bioresour Technol. 2008; 99: 297–307. 17336517 10.1016/j.biortech.2006.12.021

[31] Mauguet MC, Montillet A, Comiti J. Macrostructural characterization of granular activated carbon beds. J Mater Sci. 2005; 40: 747–755.

[32] Volesky B. Sorption and Biosorption. Montreal—St. Lambert: BV Sorbex, Inc.; 2003 pp. 233–254.

[33] Longhua X, Yuehua H, Faqin D, Hao J, Houqin W, Zhen W, et al Effects of particle size and chain length on flotation of quaternary ammonium salts onto kaolinite. Miner Petrol. 2015; 109: 309–316.

[34] Kalyani G, Rao GB, Saradhi BV, Kumar YP. Equilibrium and kinetic studies on biosorption of zinc onto *Gallus domesticus* shell powder. ARPN J Eng Appl Sci. 2009; 4: 39–49.

[35] Vinod VTP, Sashidhar RB, Sreedhar B. Biosorption of nickel and total chromium from aqueous solution by gum kondagogu (*Cochlospermum gossypium*): a carbohydrate polymer. J Hazard Mater. 2010; 178: 851–860. 10.1016/j.jhazmat.2010.02.016 20202750

[36] Benavente M, Moreno L, Martinez J. Sorption of heavy metals from gold mining wastewater using chitosan. J Taiwan Inst Chem Eng. 2011; 42: 976–998.

[37] Ho YS, McKay G. Application of kinetic models to the sorption of copper(II) on to peat. Adsorption Sci Technol. 2002; 20: 797–815.

[38] Febrianto J, Kosasih AN, Sunarso J, Ju YH, Indraswati N, Ismadji S. Equilibrium and kinetic studies in adsorption of heavy metals using biosorbent: a summary of recent studies. J Hazard Mater. 2009; 162: 616–645. 10.1016/j.jhazmat.2008.06.042 18656309

[39] Ho YS, McKay G. Pseudo-second order model for sorption processes. Process Biochem. 1999; 34: 451–465.

[40] Limousin G, Gaudet JP, Charlet L, Szenknect S, Barthès V, Krismissa M. Sorption isotherms: a review on physical bases, modeling and measurement. Appl Geochem. 2007; 22: 249–275.

[41] Padmavathy V. Biosorption of nickel(II) ions by baker´s yeast: Kinetic, thermodynamic and desorption studies. Bioresour Technol. 2008; 99: 3100–3109. 17683930 10.1016/j.biortech.2007.05.070

[42] Oliveira EA, Montanher SF, Andrade AD, Nóbrega JA, Rollemberg MC. Equilibrium studies for the sorption of chromium and nickel from aqueous solutions using raw rice bran. Process Biochem. 2005; 40: 3485–3490.

[43] Miretzky P, Saralegui A, Fernández-Cirelli A. Simultaneous heavy metal removal mechanism by dead macrophytes. Chemosphere. 2006; 62: 247–254. 15990152 10.1016/j.chemosphere.2005.05.010

[44] Pagnanelli F. Equilibrium, kinetic and dynamic modeling of biosorption processes In: Kotrba P, Mackova M, Macek T, editors. Microbial biosorption of metals. Netherlands: Springer; 2011 pp. 59–120.

[45] Vijayaraghavan K, Palanivelu K, Velan M. Treatment of nickel containing electroplating effluents with *Sargassum wightii* biomass. Process Biochem. 2006; 41: 853–859.

[46] Ghodbane I, Hamdaoui O. Removal of mercury(II) from aqueous media using eucalyptus bark: Kinetic and equilibrium studies. J Hazard Mater. 2008; 160: 301–309. 10.1016/j.jhazmat.2008.02.116 18400378

[47] León-Torres A, Cuerda-Correa E, Fernández-González C, Franco MFA, Gómez-Serrano V. On the use of a natural peat for the removal of Cr(VI) from aqueous solutions. J Colloid Interface Sci. 2012; 386: 325–332. 10.1016/j.jcis.2012.07.038 22921538

[48] Company Hach. Hach Water Analysis Handbook, 5th ed Loveland, CO; 2008.

[49] Kumar NS, Woo HS, Min K. Equilibrium and kinetic studies of 2,4,6-trichlorophenol from aqueous solutions by *Acacia leucocephala* bark. Colloids Surf B: Biointerfaces 2012; 94: 125–132. 22365378 10.1016/j.colsurfb.2012.01.048

[50] Flores-Garnica JG, Morales-Barrera L, Pineda-Camacho G, Cristiani-Urbina E. Biosorption of Ni(II) from aqueous solutions by *Litchi chinensis* seeds. Bioresour Technol. 2013; 136: 635–643. 10.1016/j.biortech.2013.02.059 23567741

[51] Khambhaty Y, Mody K, Basha S, Jha B. Kinetics, equilibrium and thermodynamic studies on biosorption of hexavalent chromium by dead fungal biomass of marine *Aspergillus niger* . Chem Eng J. 2009; 145: 489–495.

[52] Netzahuatl-Muñoz AR, Aranda-García E, Cristiani-Urbina MdC, Barragán-Huerta BE, Villegas-Garrido TL, Cristiani-Urbina E. Removal of hexavalent and total chromium from aqueous solutions by *Schinus molle* bark. Fresen Environ Bull. 2010; 19(12): 2911–2918.

[53] Elangovan R, Philip L, Chandraraj K. Biosorption of chromium species by aquatic weeds: Kinetic and mechanism studies. J Hazard Mater. 2008; 152: 100–112. 17689012 10.1016/j.jhazmat.2007.06.067

[54] Hasan SH, Singh KK, Prakash O, Talat M, Ho YS. Removal of Cr(VI) from aqueous solutions using agricultural waste `maize bran´. J Hazard Mater. 2008; 152: 356–365. 17706866 10.1016/j.jhazmat.2007.07.006

[55] Liu CC, Wang MK, Chiou CS, Li YS, Lin YA, Huang SS. Chromium removal and sorption mechanism from aqueous solutions by wine processing waste sludge. Ind Eng Chem Res. 2006; 45: 8891–8899.

[56] Plazinski W, Rudzinski W, Plazinska A. Theoretical models of sorption kinetics including a surface reaction mechanism: A review. Adv Colloid Interface Sci. 2009; 152: 2–13. 10.1016/j.cis.2009.07.009 19735907

[57] Malkoc E, Nuhoglu Y. Determination of kinetic and equilibrium parameters for the batch adsorption of Cr(VI) onto waste acorn of *Quercus ithaburensis* . Chem Eng Process. 2007; 46: 1020–1029.

[58] Suazo-Madrid A, Morales-Barrera L, Aranda-García E, Cristiani-Urbina E. Nickel(II) biosorption by *Rhodotorula glutinis* . J Ind Microbiol Biotechnol. 2011; 38: 51–64. 10.1007/s10295-010-0828-0 20820864

[59] Giles CH, Smith D, Huitson A. A general treatment and classification of the solute adsorption isotherm. I. Theoretical. J Colloid Interface Sci. 1974; 47: 755–765.

[60] Hernández-Estévez A, Cristiani-Urbina E. Nickel(II) biosorption from aqueous solutions by shrimp head biomass. Environ Monit Assess. 2014; 186: 7987–7998. 10.1007/s10661-014-3981-5 25129383

[61] Chojnacka K, Chojnacki A, Górecka H. Biosorption of Cr^3+^, Cd^2+^ and Cu^2+^ ions by blue-green algae *Spirulina* sp.: kinetics, equilibrium and the mechanism of the process. Chemosphere. 2005; 59: 75–84. 15698647 10.1016/j.chemosphere.2004.10.005

[62] Uysal M, Ar I. Removal of Cr(VI) from industrial wastewaters by adsorption. Part I: Determination of optimum conditions. J Hazard Mater. 2007; 149: 482–491. 17513041 10.1016/j.jhazmat.2007.04.019

[63] Hanif MA, Nadeem R, Bhatti HN, Ahmad NR, Ansari TM. Ni(II) biosorption by *Cassia fistula* (Golden shower) biomass. J Hazard Mater. 2007; B139: 345–355. 10.1016/j.jhazmat.2006.06.040 16860463

[64] Arıca MY, Tüzün İ, Yalçın E, İnce Ö, Bayramoğlu G. Utilisation of native, heat and acid-treated microalgae *Chlamydomonas reinhardtii* preparations for biosorption of Cr(VI) ions. Process Biochem. 2005; 40(7): 2351–2358.

[65] Cabatingan LK, Agapay RC, Rakels JLL, Otens M, Van der Wielen LAM. Potential of biosorption for the recovery of chromate in industrial wastewaters. Ind Eng Chem Res. 2001; 40: 2302–2309.

[66] González-Bermúdez Y, Rodríguez-Rico IL, Guibal E, Clero de Hoces M, Martín-Lara MA. Biosorption of hexavalent chromium from aqueous solution by *Sargassum muticum* brown alga. Application of statistical design for process optimization. Chem Eng J. 2012; 183: 68–76.

[67] Kim EJ, Park S, Hong HJ, Choi YE, Yang JW. Biosorption of chromium (Cr(III)/Cr(VI)) on the residual microalga *Nannochloris oculata* after lipid extraction for biodiesel production. Bioresour Technol. 2011; 102: 11155–11160. 10.1016/j.biortech.2011.09.107 22014703

[68] Murphy V, Hughes H, McLoughlin P. Comparative study of chromium biosorption by red, green and brown seaweed biomass. Chemosphere. 2008; 70: 1128–1134. 17884133 10.1016/j.chemosphere.2007.08.015

[69] Sari A, Tuzen M. Biosorption of total chromium from aqueous solution by red algae (*Ceramium virgatum*): equilibrium, kinetic and thermodynamic studies. J Hazard Mater. 2008; 160(2–3): 349–355. 10.1016/j.jhazmat.2008.03.005 18406520

[70] Gupta VK, Rastogi A. Biosorption of hexavalent chromium by raw and acid-treated green alga *Oedogonium hatei* from aqueous solutions. J Hazard Mater. 2009; 163(1): 396–402. 10.1016/j.jhazmat.2008.06.104 18691812

[71] Deng S, Ting YP. Polyethylenimine-modified fungal biomass as a high-capacity biosorbent for Cr(VI) anions: sorption capacity and uptake mechanisms. Environ Sci Technol. 2005; 39: 8490–8496. 16294892 10.1021/es050697u

[72] Fiol N, Villaescusa I, Martínez M, Miralles N, Poch J, Serrarlos J. Biosorption of Cr(VI) using low cost sorbents. Environ Chem Lett. 2003; 1: 135–139.

[73] Cimino G, Passerini A, Toscano G. Removal of toxic cations and Cr(VI) from aqueous solution by hazelnut shell. Water Res. 2000; 34(11): 2955–2962.

[74] Niu H, Volesky B. Biosorption of chromate and vanadate species with waste crab shells. Hydrometallurgy. 2006; 84(1–2): 28–36.

[75] Nakajima A, Baba Y. Mechanism of hexavalent chromium adsorption by persimmon tannin gel. Water Res. 2002; 38(12): 2859–2864. 10.1016/j.watres.2004.04.005 15223280

[76] Altundogan HS. Cr(VI) removal from aqueous solution by iron (III) hydroxide-loaded sugar beet pulp. Process Biochem. 2005; 40: 1443–1452.

[77] Liu SX, Chen X, Chen XY, Liu ZF, Wang HL. Activated carbon with excellent chromium(VI) adsorption performance prepared by acid—base surface modification. J Hazard Mater. 2007; 141: 315–319. 16914264 10.1016/j.jhazmat.2006.07.006

[78] Lopez-Nuñez PV, Aranda-García E, Cristiani-Urbina MdC, Morales-Barrera L, Cristiani-Urbina E. Removal of hexavalent and total chromium from aqueous solutions by plum (*P*. *domestica* L.) tree bark. Environ Eng Manag J. 2014; 13: 1927–1938.

[79] Matouq M, Jildeh N, Qtaishat M, Hindiyeh M, Al Syouf MQ. The adsorption kinetics and modeling for heavy metals removal from wastewater by *Moringa* pods. J Environ Chem Eng. 2015; 3: 775–784.

[80] Zhao J, Zhang X, He X, Xiao M, Zhang W, Lu C. A super biosorbent from dendrimer poly(amidoamine)-grafted cellulose nanofibril aerogels for effective removal of Cr(VI). J Mater Chem A 2015; 3: 14703–14711.

[81] Aksu Z. Determination of the equilibrium, kinetic and thermodynamic parameters of the batch biosorption of nickel(II) ions onto *Chlorella vulgaris* . Process Biochem. 2002; 38: 89–99.

[82] Pandey PK, Sharma SK, Sambi SS. Kinetics and equilibrium study of chromium adsorption on zeoliteNaX. Int J Environ Sci Technol. 2010; 7: 395–404.

[83] Guerrero-Coronilla I, Morales-Barrera L, Cristiani-Urbina E. Kinetic, isotherm and thermodynamic studies of amaranth dye biosorption from aqueous solution onto water hyacinth leaves. J Environ Manag. 2015; 152: 99–108. 10.1016/j.jenvman.2015.01.026 25617874

[84] Fathy NA, El-Wakeel ST, El-Latif RRA. Biosorption and desorption studies on chromium(VI) by novel biosorbents of raw rutin and rutin resin. J Environ Chem Eng. 2015; 3: 1137–1145.

[85] Liang FB, Song YL, Huang CP, Zhang J, Chen BH. Adsorption of hexavalent chromium on a lignin-based resin: Equilibrium, thermodynamics, and kinetics. J Environ Chem Eng. 2013; 1: 1301–1308.

